# Supplementary material for: Cytokine Profiling of Children, Adolescents, and Young Adults Newly Diagnosed with Sarcomas Demonstrates the Role of IL-1β in Osteosarcoma Metastasis
Source: Cancers (Basel). 2025 Sep 15;17(18):3009. doi: 10.3390/cancers17183009 (PMC12468999; doi:10.3390/cancers17183009)
Supplement: Supplementary file 1 [file cancers-17-03009-s001.zip › cancers-3833777-supplementary.pdf]

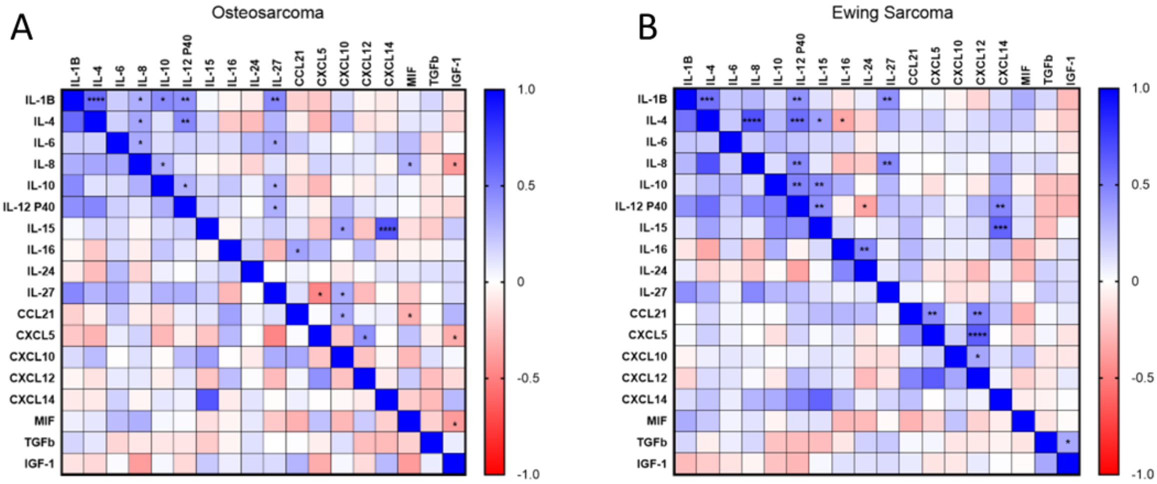

Supplementary Figure S1. Correlation matrix of spearman correlation coefficients indicated by color gradient for each analyte in patients with (A) Osteosarcoma and (B) Ewing Sarcoma. Asterisks designate significance for that relationship where *, **, ***, and **** denote p<0.05, p<0.01, p<0.001, p<0.0001. Where no asterisks are shown, correlation was not statistically significant.

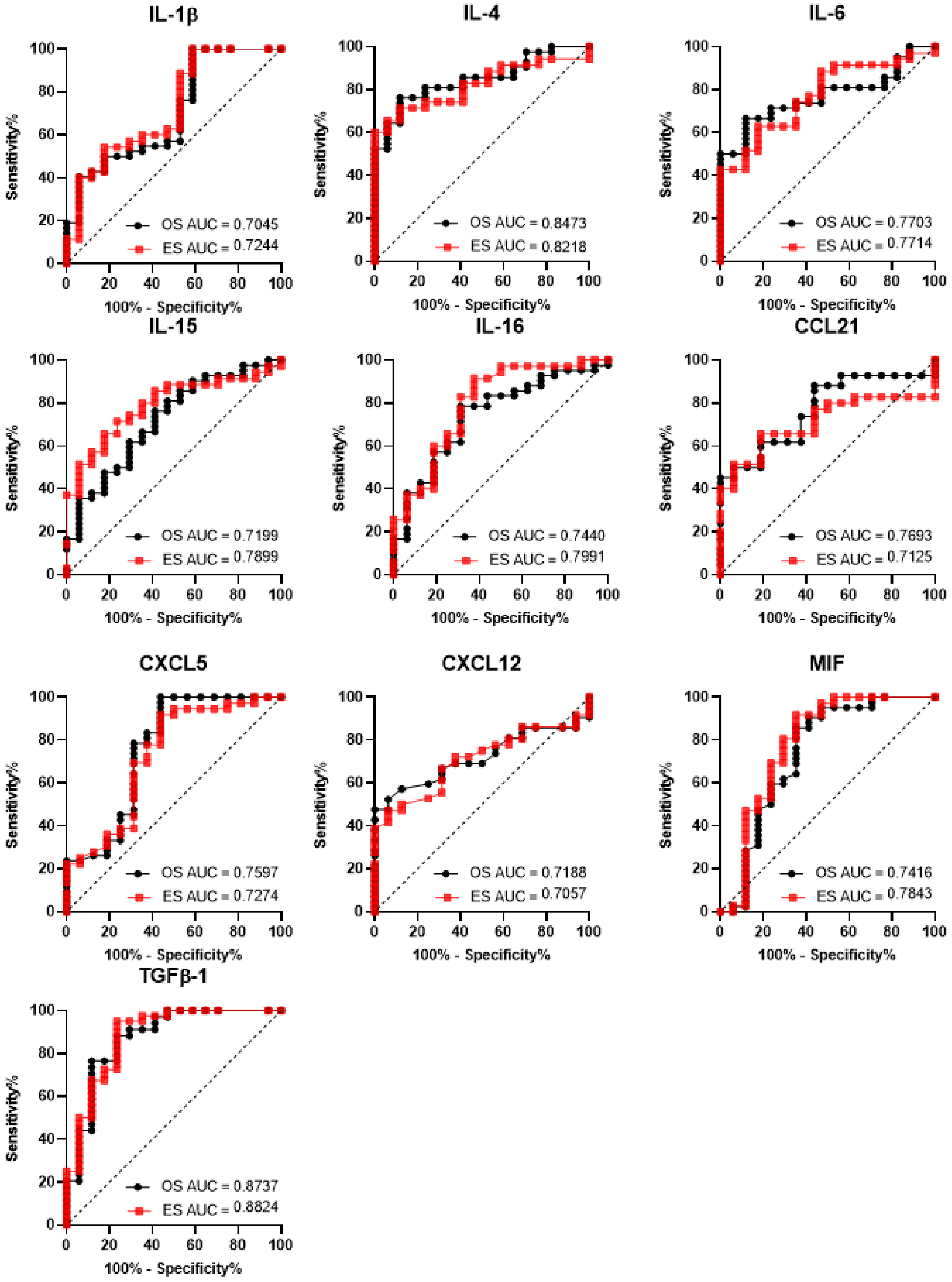

Supplementary Figure S2. Receiver operating characteristics curves and area under the curve (AUC) for each cytokine that was significantly different in at least one sarcoma cohort compared to the healthy cohort. Curves compare healthy to osteosarcoma (OS) in black and Ewing sarcoma (ES) in red

Cohort	Healthy	Ewing Sarcoma	Fusion Positive Rhabdomyosarcoma	Fusion Negative Rhabdomyosarcoma	Osteosarcoma	Other Sarcoma
Age, n (%)						
≤21	1 (5.9%)	29 (80.6%)	8 (100%)	22 (91.7%)	38 (90.5%)	9 (69.2%)
≥22	16 (94.1%)	7 (19.4%)	0 (0%)	2 (8.3%)	4 (9.5%)	4 (30.8%)
Gender, n (%)						
Female	7 (41.2%)	18 (50.0%)	6 (75.0%)	11 (45.8%)	20 (47.6%)	6 (46.2%)
Male	10 (58.8%)	18 (50.0%)	2 (25.0%)	13 (54.2%)	22 (52.4%)	7 (53.8%)
Stage, n (%)						
Localized		25 (73.5%)	6 (75.7%)	21 (91.3%)	36 (87.8%)	9 (75.0%)
Metastatic		11 (26.5%)	1 (14.3%)	2 (8.7%)	5 (12.2%)	4 (25.0%)

Supplementary Table S1: Demographic characteristics of subjects included in this analysis. Not all patients had stage recorded.

Analyte		IL-1β pg/mL	IL-4 pg/mL	IL-6 pg/mL	IL-8 pg/mL	IL-10 pg/mL	IL-16 pg/mL	TGFβ-1 pg/mL	IGF-1 pg/mL
Healthy		0.99 [0.25-1.08]	0.13 [-0.13-0.27]	0.19 [0.08-0.42]	0.5 [0.33-0.78]	1.14 [0.65-1.35]	2.99 [2.55-3.13]	4.94 [1.82-5.08]	4.58 [4.33-4.80]
Ewing sarcoma		1.11 [0.96-1.23]	0.61 [0.23-0.77]	0.56 [0.32-0.92]	0.66 [0.53-0.9]	1.11 [0.77-1.32]	2.47 [2.05-2.76]	4.25 [3.63-4.74]	4.40 [4.21-4.66]
	p	**	***	***	NS	NS	***	****	NS
Fusion positive Rhabdomyosarcoma		1.17 [0.79-1.62]	0.76 [0.29-0.86]	0.68 [-0.09-1.3]	0.74 [0.58-1.05]	1.02 [0.74-1.69]	2.21 [1.84-2.78]	4.80 [4.66-5.22]	4.57 [4.24-4.92]
	p	*	**	NS	NS	NS	**	*	NS
Fusion Negative Rhabdomyosarcoma		1.12 [0.87-1.33]	0.46 [0.19-0.76]	0.66 [0.53-0.9]	0.62 [0.38-0.97]	0.99 [0.64-1.33]	2.53 [2.2-2.8]	4.94 [4.83-5.06]	4.41 [3.93-4.73]
	p	*	**	***	NS	NS	**	****	NS
Osteosarcoma		1.09 [0.88-1.32]	0.5 [0.31-0.77]	0.62 [0.29-0.95]	0.7 [0.52-0.89]	1 [0.73-1.33]	2.45 [2.26-2.79]	4.94 [4.80-5.09]	4.70 [4.35-4.94]
	p	**	****	***	NS	NS	***	****	NS
Other Sarcoma		1.08 [0.94-1.49]	0.59 [0.27-0.85]	0.56 [-0.2-0.66]	0.62 [0.35-0.71]	0.92 [0.76-1.5]	2.53 [2.35-2.72]	4.86 [4.73-4.94]	4.61 [4.45-4.84]
	p	*	***	NS	NS	NS	*	**	*

Analyte		CXCL5 pg/mL	CXCL10 pg/mL	CXCL12 pg/mL	CXCL14 pg/mL	MIF pg/mL
Healthy		2.38 [2.04-3.18]	1.97 [1.81-2.16]	3.21 [3.05-3.33]	2.51 [2.42-3.55]	2.14 [1.61-2.48]
Ewing sarcoma		2.95 [2.71-3.35]	2.05 [1.75-2.2]	3.34 [3.2-3.48]	3.39 [3.2-3.52]	2.55 [2.41-2.69]
	p	**	NS	*	NS	NS
Fusion positive Rhabdomyosarcoma		2.98 [2.74-3.26]	2.08 [1.9-2.27]	3.22 [2.96-3.28]	3.29 [3.17-3.71]	2.48 [2.46-2.79]
	p	NS	NS	NS	NS	NS
Fusion Negative Rhabdomyosarcoma		3 [2.84-3.25]	2.09 [1.9-2.26]	3.29 [3.21-3.39]	3.33 [3.22-3.47]	2.49 [2.4-2.6]
	p	*	NS	*	*	**
Osteosarcoma		3.03 [2.76-3.35]	2.07 [1.94-2.27]	3.37 [3.18-3.48]	3.4 [3.27-3.47]	2.5 [2.35-2.65]
	p	**	NS	*	NS	***
Other Sarcoma		2.86 [2.75-3.39]	2 [1.92-2.11]	3.36 [3.28-3.51]	3.35 [3.27-3.66]	2.61 [2.41-2.82]
	p	*	NS	*	*	**

Analyte		IL-12 P40 pg/mL	IL-15 pg/mL	IL-24 pg/mL	IL-27 pg/mL	CCL21 pg/mL
Healthy		1.95 [1.84-2.06]	0.98 [0.74-1.06]	1.73 [1.54-2.07]	2.72 [2.57-2.97]	2.68 [2.63-2.78]
Ewing sarcoma		1.9 [1.73-2.08]	0.66 [0.25-0.87]	1.87 [1.55-2.15]	2.79 [2.6-2.88]	2.87 [2.73-3.01]
	p	NS	***	NS	NS	*
Fusion positive Rhabdomyosarcoma		2.01 [1.85-2.08]	0.81 [0.55-1.25]	1.91 [1.53-2.13]	2.69 [2.62-2.99]	2.8 [2.62-2.9]
	p	NS	NS	NS	NS	NS
Fusion Negative Rhabdomyosarcoma		1.98 [1.57-2.1]	0.7 [0.39-0.81]	2.03 [1.84-2.28]	2.61 [2.46-2.79]	2.91 [2.81-3.02]
	p	NS	**	NS	NS	***
Osteosarcoma		1.97 [1.85-2.09]	0.76 [0.56-0.94]	1.87 [1.58-2.08]	2.64 [2.53-2.75]	2.89 [2.75-2.97]
	p	NS	****	NS	NS	*
Other Sarcoma		1.83 [1.55-2.12]	0.79 [0.5-1.07]	2.06 [1.56-2.39]	2.74 [2.49-2.87]	2.84 [2.77-2.96]
	p	NS	NS	NS	NS	*

Supplementary Table S2: Cytokine concentrations in plasma of healthy subjects and sarcoma patients at diagnosis. Median [IQR]. Statistical comparison of cohort to healthy controls was performed using the Mann-Whitney test where *, **, ***, and **** denote $p<0.05$, $p<0.01$, $p<0.001$, $p<0.0001$

Clinicopathological Parameter		IL-1β pg/mL	IL-4 pg/mL	IL-6 pg/mL	IL-8 pg/mL	IL-10 pg/mL	IL-16 pg/mL	CXCL5 pg/mL	CXCL10 pg/mL	CXCL12 pg/mL	CXCL14 pg/mL	MIF pg/mL	TGFβ-1 pg/mL	IGF-1 pg/mL
Age														
Mixed Cohorts	≤21	1.10	0.51	0.64	0.67	1.06	2.68	3.01	1.92	3.33	3.37	2.49	4.93	4.61
	≥22	1.11	0.66	0.29	0.62	0.90	2.75	2.88	1.83	3.35	3.39	2.67	4.95	4.58
	p	*0.39*	*0.42*	***0.02***	*0.77*	*0.53*	***0.044***	*0.31*	*0.75*	*0.58*	*0.98*	***0.044***	*0.61*	*0.82*
Ewing sarcoma	≤21	1.13	0.51	0.58	0.65	1.13	2.53	2.95	2.04	3.34	3.40	2.50	4.92	4.58
	≥22	1.04	0.64	0.28	0.84	0.91	2.33	3.28	2.10	3.36	3.40	2.83	5.03	4.70
	p	*0.85*	*0.39*	*0.87*	*0.46*	*0.22*	*0.058*	*0.58*	*0.53*	*0.72*	*0.98*	***0.018***	*0.19*	*0.43*
Osteo-sarcoma	≤21	1.02	0.45	0.63	0.71	1.00	2.47	3.05	2.07	3.38	3.40	2.50	4.94	4.73
	≥22	1.21	0.70	0.14	0.59	1.17	2.23	2.74	2.15	3.27	3.41	2.46	4.94	4.51
	p	*0.25*	*0.44*	*0.41*	*0.59*	*0.69*	*0.21*	*0.47*	*0.39*	*0.96*	*0.84*	*0.92*	*0.65*	*0.23*
Stage														
Mixed Cohorts	Localized	1.10	0.54	0.59	0.68	0.99	2.67	2.99	1.96	3.33	2.50	3.38	4.93	4.61
	Metastatic	1.23	0.66	0.63	0.71	1.10	2.75	2.89	1.81	3.29	2.53	3.39	4.93	4.50
	p	***0.022***	*0.26*	*0.51*	*0.94*	*0.34*	*0.36*	*0.86*	*0.58*	*0.27*	*0.92*	*0.20*	*0.59*	*0.40*
Ewing sarcoma	Localized	1.07	0.60	0.49	0.65	1.12	2.46	2.95	2.00	3.33	3.41	2.54	4.94	4.68
	Metastatic	1.21	0.69	0.75	0.76	1.02	2.51	3.42	2.06	3.38	3.40	2.50	4.93	4.33
	p	*0.060*	*0.36*	*0.20*	*0.49*	*0.65*	*0.36*	*0.26*	*0.58*	*0.90*	*0.96*	*0.70*	*0.59*	*0.061*
Osteo-sarcoma	Localized	1.05	0.54	0.62	0.70	0.96	2.43	3.05	2.07	3.40	3.40	2.50	4.93	4.70
	Metastatic	1.32	0.48	0.60	0.83	1.25	2.45	2.88	2.26	3.20	3.40	2.45	5.04	4.98
	p	*0.068*	*0.65*	*0.74*	*0.77*	*0.30*	*0.59*	*0.33*	*0.20*	*0.11*	*0.58*	*0.90*	*0.58*	*0.16*

Clinicopathological Parameter		IL-12p40 pg/mL	IL-15 pg/mL	IL-24 pg/mL	IL-27 pg/mL	CCL21 pg/mL
Age						
Mixed Cohorts	≤21	1.97	0.69	2.07	2.88	2.52
	≥22	1.84	0.80	2.06	2.77	2.41
	p	*0.23*	*0.14*	*0.44*	*0.29*	*0.065*
Ewing sarcoma	≤21	1.84	0.65	1.95	2.78	2.90
	≥22	1.98	0.67	1.54	2.85	2.83
	p	*0.75*	*0.64.*	*0.069*	*0.58*	*0.65*
Osteo-sarcoma	≤21	1.97	0.73	1.89	2.63	2.93
	≥22	2.00	0.91	1.64	2.79	2.75
	p	*0.77*	*0.49*	*0.12*	*0.43*	***0.0082***
Stage						
Mixed Cohorts	Localized	1.96	0.70	2.06	2.89	2.48
	Metastatic	1.96	0.75	2.06	2.84	2.57
	p	*0.56*	*0.41*	*0.35*	*0.16*	*0.44*
Ewing sarcoma	Localized	1.84	0.65	1.97	2.78	2.90
	Metastatic	1.98	0.67	1.81	2.75	2.84
	p	*0.18*	*0.98*	*0.37*	*0.17*	*0.31*
Osteo-sarcoma	Localized	1.97	0.73	1.86	2.63	2.89
	Metastatic	2.08	0.89	1.99	2.75	2.96
	p	*0.38*	*0.19*	*0.71*	*0.15*	*0.49*

Supplementary Table S3. Association Between Cytokine Level and Clinicopathological Parameters. Median values displayed with significance determined by Mann-Whitney test. Hazard ratio of high to low group for overall survival (OS) and event free survival (EFS) by log rank test. P values ≤ 0.05 notated in bold. Values rounded to 2 significant figures.

Survival		Hazard ratio (high/low) IL-1β	IL-4	IL-6	IL-8	IL-10	IL-16	CXCL5	CXCL10	CXCL12	CXCL14	MIF	TGFβ-1	IGF-1
Mixed Cohorts	EFS	1.2	1.3	1.3	0.93	0.84	0.99	1.1	1.0	1.1	1.1	1.1	0.98	1.1
	p	*0.86*	*0.16*	*0.087*	*0.67*	*0.057*	*0.91*	*0.69*	*0.92*	*0.46*	*0.50*	*0.51*	*0.90*	*0.66*
	OS	1.0	1.1	1.1	1.0	0.79	1.1	1.2	0.85	1.4	1.0	1.0	1.0	0.92
	p	*0.23*	*0.39*	*0.44*	*0.87*	*0.32*	*0.35*	*0.076*	*0.21*	***0.0040***	*0.96*	*0.85*	*0.78*	*0.49*
Ewing sarcoma	EFS	1.3	1.0	1.2	0.96	0.99	1.3	0.79	1.3	1.2	1.4	0.83	0.76	1.2
	p	*0.85*	*0.98*	*0.38*	*0.86*	*0.97*	***0.028***	*0.27*	*0.34*	*0.52*	*0.31*	*0.63*	*0.19*	*0.48*
	OS	1.0	1.1	1.3	1.1	1.1	1.3	1.1	0.63	1.3	1.0	0.80	1.1	1.0
	p	*0.82*	*0.76*	*0.19*	*0.76*	*0.79*	*0.18*	*0.78*	***0.049***	*0.22*	*0.87*	*0.34*	*0.73*	*0.88*
Osteo-sarcoma	EFS	2.4	1.8	1.6	1.0	0.89	1.2	1.3	0.95	0.84	1.0	1.2	0.83	1.0
	p	***0.0097***	*0.058*	*0.14*	*0.96*	*0.73*	*0.56*	*0.36*	*0.83*	*0.52*	*0.99*	*0.58*	*0.58*	*0.89*
	OS	1.5	1.4	1.4	0.96	0.89	1.4	1.7	0.96	1.2	1.3	1.3	1.2	0.72
	p	*0.30*	*0.22*	*0.22*	*0.86*	*0.66*	*0.15*	***0.010***	*0.85*	*0.29*	*0.29*	*0.25*	*0.42*	*0.11*

Survival		Hazard ratio (high/low) IL-12p40	IL-15	IL-24	IL-27	CCL21
Mixed Cohorts	EFS	1.0	1.1	1.3	0.93	0.84
	p	*0.98*	*0.52*	*0.15*	*0.67*	*0.91*
	OS	1.0	0.85	0.95	1.0	1.0
	p	*0.77*	*0.20*	*0.65*	*0.95*	*0.35*
Ewing sarcoma	EFS	0.98	1.1	1.2	0.97	0.67
	p	*0.93*	*0.87*	***0.03***	*0.89*	*0.20*
	OS	1.1	0.84	1.3	1.1	0.72
	p	*0.79*	*0.4*	*0.16*	*0.79*	*0.096*
Osteo-sarcoma	EFS	1.5	1.2	2.6	1.7	1.0
	p	*0.15*	*0.46*	***0.018***	*0.071*	*0.86*
	OS	1.3	1.2	0.40	0.88	1.2
	p	*0.24*	*0.38*	***0.046***	*0.71*	*0.26*

Supplementary Table S4. Hazard ratio of high to low group for overall survival (OS) and event free survival (EFS) by log rank analysis. P values ≤ 0.05 notated in bold. Values rounded to 2 significant figures.
